# Mechanisms of resistance to RET-directed therapies

**DOI:** 10.1530/ERC-24-0224

**Published:** 2025-01-10

**Authors:** Roderick J Clifton-Bligh

**Affiliations:** Cancer Genetics, Kolling Institute, Royal North Shore Hospital and University of Sydney, Sydney, New South Wales, Australia

**Keywords:** RET, medullary thyroid cancer, selpercatinib, pralsetinib

## Abstract

The association between *RET* and multiple endocrine neoplasia type 2 was established in 1993 and remains one of the very few oncogenes for which distinct phenotypes (medullary thyroid cancer or pheochromocytoma) are associated with the same hot-spot variants occurring in either germline or somatic DNA. Somatic *RET* fusion events have also been described in several cancers, including papillary thyroid cancer, non-small-cell lung cancer, breast cancer, salivary gland cancer and pancreatic cancer. Highly selective RET inhibitors have improved outcomes in *RET*-altered cancers and have been well-tolerated. Nevertheless, primary and acquired drug resistance has been observed, arising from distinct genomic alterations either in *RET* (on-target resistance) or via alternate oncogenic pathways (bypass resistance). The same mechanisms of resistance have been observed across multiple cancer types, which implies *RET*-altered cancers evolve away from RET addiction via stochastic subclonal events. Understanding these mechanisms is crucial for identifying therapeutic opportunities to overcome resistance. Successful treatment targeting bypass oncogenes has been reported in several instances, at least for short-term outcomes; in contrast, although several compounds have been reported to overcome on-target *RET* alterations, none have yet been translated into routine clinical practice and this remains an area of urgent clinical need.

## Introduction

After the cloning of rearranged during transfection (*RET*) proto-oncogene on chromosome 10q11.2 (Takahashi *et al.* 1985), its association with various human malignancies was progressively uncovered: first, with finding somatic *RET* rearrangements (as fusion genes) in up to 20% papillary thyroid cancers (PTC) (Grieco *et al.* 1990, Viglietto *et al.* 1995) and then establishing *RET* germline mutations as the cause of multiple endocrine neoplasia type 2A (MEN2A) (Donis-Keller *et al.* 1993, Mulligan *et al.* 1993) and MEN2B (Carlson *et al.* 1994, Eng *et al.* 1994, Hofstra *et al.* 1994). Since then, hot-spot mutations in *RET* exons 8, 10, 11 and 13–16 and rarely 1 and 5 have been found in nearly all cases of MEN2A and 2B syndromes (Gild *et al.* 2023). Remarkably, the same mutations (most commonly M918T; this review will use amino acid terminology commonly used by clinicians throughout, i.e. M918T instead of p.Met918Thr) also occur somatically in around 25–50% of sporadic medullary thyroid cancers (MTC) (Romei *et al.* 2016). Somatic *RET* rearrangements (as fusion genes with *CCDC6*, *NCOA4*, *KIF5B*, *PRKAR1A*, *TRIM33* and other rarer partners) have now been found in 1–2% of non-small-cell lung cancers (NSCLC) and at lower prevalence in many other cancers including pancreatic, colon, breast and salivary gland (Ou & Zhu 2020). Intriguingly, *RET* fusions also develop as a mechanism of acquired resistance to EGFR inhibitor treatment in NSCLC (Piotrowska *et al.* 2018, Wang *et al.* 2022).

**Figure 1:**
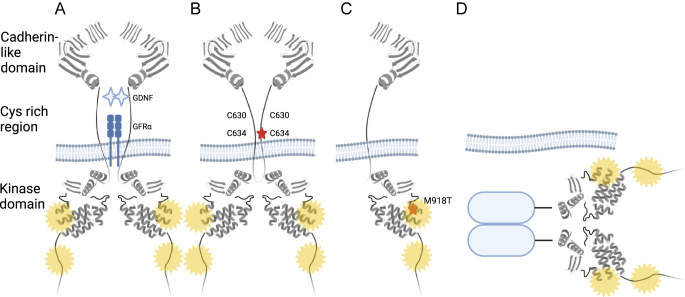
Normal and pathological RET signaling: (A) RET is normally activated after binding of glial cell line-derived neurotrophic factor (GDNF) ligand dimers to GDNF family receptor-alpha (GFRα) co-receptors, triggering transautophosphorylation of tyrosine residues in the RET cytoplasmic domain (*starbursts*), (B) mutations in *RET* cysteine-rich domain associated with MTC lead to ligand-independent homodimerization *via* interchain disulfide bridges, (C) mutations at *RET* M918T or A883F associated with MTC lead to monomeric activation of the kinase domain and (D) *RET* fusion partners associated with PTC, non-small-cell lung cancers or other cancers provide a dimerization domain for RET homodimers to form intracellularly and activate downstream signaling cascades. Data from Drilon *et al.* 2018*a*,*b*, Hu *et al.* 2023. Created with BioRender.com.

The protein RET is a single transmembrane tyrosine kinase, which normally forms a cell-surface homodimer complex, which on binding glial-derived neurotrophic factor family neurotrophins triggers transautophosphorylation of tyrosine residues in the intracellular kinase domain (Manie *et al.* 2001) (Fig. 1A). These phosphotyrosines then recruit several adaptor proteins to mediate the activation of downstream signaling cascades including phospholipase C*γ*/protein kinase C, c-Jun N-terminal kinases, Src-related kinases and nuclear factor *κ*B, P13 kinase/AKT and the MAP kinase pathways (Li *et al.* 2019).

*RET* mutations cause constitutive kinase activation by one of the three main mechanisms (Drilon *et al.* 2018*a*): (a) ligand-independent homodimerization via interchain disulfide bonds (classically seen in *RET* mutations at cysteine residues in codons 610 and 634; Fig. 1B), (b) monomeric activation of the kinase domain (e.g., *RET* M918T and A883F; Fig. 1C) and (c) for *RET* fusions, dimerization or oligomerization via coiled-coil or leucine-zipper domains in the fusion partner (Fig. 1D). *RET* gain-of-function by amplification is also seen in a few cancer types (Kato *et al.* 2017).

This strong genotype–phenotype relationship has driven the search for RET kinase inhibitors for treating RET-altered cancers. These inhibitors have targeted mainly the ATP-binding pocket within the catalytic domain (Santoro & Carlomagno 2006). Modest success of multikinase inhibitors (MKIs) paved the way for more selective RET inhibitors, which have now become first-line therapies for RET-altered cancers ((Kurzrock *et al.* 2011), Wells *et al.* 2012, Wirth *et al.* 2020). However, these successes have been moderated by the emergence of drug resistance, which continues to challenge clinical practice and inform further efforts in novel drug design.

This review examines mechanisms of resistance to RET inhibitors in the context of their discovery in clinical cases and experimental systems, including on-target and bypass mechanisms but also sanctuary sites (brain) and dose-limiting tolerability. The focus will be on clinical trials of RET inhibitors in thyroid cancer, drawing on the extensive insights gained from their use in NSCLC.

## Defining resistance

Drug resistance is defined as the progression of disease despite maximally tolerated doses. Resistance may be primary or acquired; primary resistance is defined where best oncological response is progressive disease, whereas acquired resistance is defined when progressive disease occurs after a period of objective response. Mechanisms of resistance can be divided broadly into on-target (variation in the target that prevents drug response), off-target or bypass (activation of an alternate oncogenic pathway), or reduced drug accumulation at the target site (either by the lack of penetration (e.g., cerebral) or by the upregulation of drug-efflux pumps) (Lin & Shaw 2016). Lineage plasticity – where adenocarcinomas develop resistance through epithelial-to-mesenchymal transition, seen in some cases of NSCLC treated with EGFR inhibitors (Schoenfeld *et al.* 2020) – has been reported in two recent cases of resistance to RET inhibition (Gazeu *et al.* 2023, Peng *et al.* 2023) although this mechanism of resistance does not appear to be common (Lin *et al.* 2020, Rosen *et al.* 2022). Progressive dedifferentiation and increased proliferative rate has been noted in subjects who developed resistance to RET inhibition (Hadoux *et al.* 2023b).

To confirm a mechanism of resistance, it is not sufficient for an oncogenic variant to be present simply at the time resistance emerges; for instance, PI3K signaling activation is not uncommonly observed in RET-altered cancers but does not appear to be associated with resistance to RET inhibitors (Rosen *et al.* 2022). Rather, the putative resistance mechanism must be shown to correlate with disease progression across multiple sites of disease and in multiple patients, be validated in experimental systems and, ideally, be shown to respond to retargeted therapy where available.

Discovering mechanisms of resistance to targeted therapies has accelerated rapidly through routine use of next-generation sequencing (NGS), initially on tissue biopsies but more recently using liquid biopsies of cell-free and circulating tumor DNA (ctDNA), which can monitor emergence of a range of oncogenic pathways including genetic variation in the target at reasonable sensitivity. Limitations of liquid biopsy need also to be considered – limit of detection is generally around 1% variant allele frequency, which may miss resistance variants at an early stage, and NGS panels may not include all possible variations associated with resistance (Subbiah *et al.* 2021d). Nevertheless, the noninvasive nature of liquid biopsies has facilitated personalized cancer care adapted to treatment-emergent resistance pathways.

## RET inhibition: MKIs

Similarity of the ATP-binding domain in many tyrosine kinase receptors led to the therapeutic development of MKIs with activity against multiple targets. Initial MKIs were developed focusing on the inhibition of VEGF receptors with vandetanib emerging as a highly potent inhibitor of Flt-1 (VEFGR-1) and KDR (VEGFR-2) and of RET kinase activity including oncogenic RET mutants and fusion isoforms (Carlomagno *et al.* 2002, Vidal *et al.* 2005). In the ZETA trial, patients with locally advanced or metastatic MTC treated with vandetanib had superior progression-free survival (PFS) compared to placebo (19.3 vs 30.5 months, respectively; hazard ratio (HR) 0.46; 95% CI 0.31–0.69) (Wells *et al.* 2012). Vandetanib received FDA approval for treatment of non-resectable advanced or metastatic MTC in 2011.

Cabozantinib is a potent inhibitor of c-MET, VEGFR2 and other kinases including RET, FLT3, TRK and KIT (Yakes *et al.* 2011). In patients with progressive MTC in the EXAM study, cabozantinib improved PFS compared to placebo (11.2 vs 4 months), although objective response rate (ORR) was only 28% (Elisei *et al.* 2013). The median overall survival after long-term follow-up (minimum 42 months) in MTC harboring *RET* M918T on cabozantinib was 44.3 vs 18.9 months for those on placebo (HR, 0.60; 95% CI, 0.38–0.94) (Schlumberger *et al.* 2017). Treatment-related adverse events were frequent and often dose-limiting including diarrhea, fatigue, hypertension and other toxicities (Elisei *et al.* 2013). Cabozantinib was FDA-approved for treatment of MTC in 2012. Cabozantinib has also been studied in other RET-altered cancers including renal cell carcinoma (Choueiri *et al.* 2014) and NSCLC (Drilon *et al.* 2016).

Other MKIs have been studied in RET-altered cancers including lenvatinib, which had modest success in MTC with ORR 36% and PFS of 9 months (Schlumberger *et al.* 2016). Lenvatinib was FDA-approved for radioiodine-refractory differentiated thyroid cancer in 2015, some of which cases will be associated with *RET* fusions (Schlumberger *et al.* 2015).

Dose-limiting tolerability of these MKIs emphasizes two points: first, since tolerated doses are often below that required for complete RET inhibition, their efficacy may in fact depend on inhibiting VEGF receptors or other kinases; second, incomplete RET inhibition may increase the risk of treatment resistance emerging (Tiedje & Fagin 2020). Nonetheless, these MKIs remain the cornerstone of managing patients with non-RET-driven advanced, progressive MTC.

Early pre-clinical data showed naturally occurring *RET* V804L/M gatekeeper mutations conferred resistance to vandetanib *via* steric hindrance to drug binding (Carlomagno *et al.* 2004). Notably, these mutations occur spontaneously at the germline or somatic level in MEN2A or sporadic MTC, respectively, and cause constitutive ligand-independent activation of RET signaling (Iwashita *et al.* 1999). Subsequently, these gatekeeper mutations were shown to emerge in patients with *RET*-altered MTC or NSCLC after treatment with either vandetanib or cabozantinib (Dagogo-Jack *et al.* 2018, Subbiah *et al.* 2018*a*,*b*, Wirth *et al.* 2019) (Supplementary Table (see the section on Supplementary materials given at the end of the article)). Another mechanism of vandetanib resistance was reported in a patient with NSCLC and *CCDC6-RET*, in which acquisition of *RET* S904F resulted in increased basal kinase activity (Nakaoku *et al.* 2018).

Other non-gatekeeper *RET* mutations may be resistant to these MKIs; *RET* D898_E901del was shown to be insensitive to cabozantinib and partially sensitive to vandetanib (Porcelli *et al.* 2023). Nevertheless, some patients with MTC associated with this variant had short-term tumor control on cabozantinib possibly due to its antiangiogenic effects (Porcelli *et al.* 2023).

Some MKIs including vandetanib may have poor cerebral penetration leading to low response rates in patients with brain metastases (Drilon *et al.* 2018*a*,*b*).

## RET inhibition: highly specific inhibitors

First-generation selective inhibitors were designed to bind the RET kinase domain but avoiding steric clash with the gatekeeper V804L/M mutations described above. Unlike MKIs, which access the front and back clefts of RET kinase domain via the ‘gate’ at V804, selpercatinib (LOXO-292) and pralsetinib (BLU-667) avoid the gate by a wrap-around access to front and back clefts (Subbiah *et al.* 2021*a*). These drugs are also far more selective for the RET kinase, with less off-target kinase inhibition and therefore better tolerability (Brandhuber *et al.* 2016).

Promising preclinical and early clinical studies of these two selective RET kinase inhibitors (Subbiah *et al.* 2018, Wirth *et al.* 2019) led to phase 1/2 trials LIBRETTO-001 (selpercatinib) (Drilon *et al.* 2020*a*, Wirth *et al.* 2020) and ARROW (pralsetinib) (Gainor *et al.* 2021*a*, Subbiah *et al.* 2021*b*). These clinical trials recruited patients who were naïve to MKIs, and those who had been pre-treated. Both selpercatinib and pralsetinib showed durable responses not only in *RET*-altered thyroid cancers and NSCLCs but also in other malignancies associated with *RET* mutations (Drilon *et al.* 2020*a*, Wirth *et al.* 2020, Gainor *et al.* 2021*a*, Subbiah *et al.* 2021*b*, 2022*a*,*b*). For instance, in treatment-naïve MTC, ORR was 73% for selpercatinib and 71% for pralsetinib; in pretreated MTC, ORR was 69 and 60%, respectively; and in *RET* fusion PTC, ORR was 79 and 89%, respectively (Wirth *et al.* 2020, Subbiah *et al.* 2021*b*). Median PFS rates are not yet defined although complete responses were uncommon. Cerebral penetration was also evident for each inhibitor with responses of intracranial metastases from NSCLC (Drilon *et al.* 2020*a*, Gainor *et al.* 2021*a*). Treatment-related adverse events were usually mild and most commonly hypertension, elevated transaminases and fatigue/asthenia; more serious but less common adverse events included hypersensitivity or QT prolongation (selpercatinib) and pneumonitis or anemia/neutropenia (pralsetinib). Dose reductions for toxicity were seen in 30% of patients on selpercatinib and 46% on pralsetinib, with discontinuation rates of only 2–4% for either (Wirth *et al.* 2020, Subbiah *et al.* 2021*b*). Efficacy of these two targeted treatments despite previous MKI therapy again emphasizes the importance of achieving maximal inhibition of RET kinase when treating these cancers. Selpercatinib is now being studied in neoadjuvant settings for advanced thyroid cancers (NCT04759911).

Recently, results from a phase 3 randomized trial (LIBRETTO-531) reported superior progression-free survival with selpercatinib as compared with physician’s choice of either cabozantinib or vandetanib in treatment-naive patients with progressive, advanced *RET* mutant medullary thyroid cancer; hazard ratio for disease progression or death was 0.28 (95% CI, 0.16–0.48; *P* < 0.001), and PFS at 12 months was 86.8% (95% CI, 79.8–91.6) in the selpercatinib group and 65.7% (95% CI, 51.9–76.4) in the control group (Hadoux *et al.* 2023*a*). ORR was 69.4% (95% CI, 62.4–75.8) in the selpercatinib group – including 11.9% with complete responses – and 38.8% (95% CI, 29.1–49.2) in the control group. Treatment discontinuation was only 4.7% in the selpercatinib arm compared to 26.8% in the control (cabozantinib or vandetanib) arm.

Importantly, both selpercatinib and pralsetinib efficacy appears independent of the mechanism by which RET is activated – response rates have been similar in cancers containing *RET* M918T, other missense *RET* variants associated with MEN2A and *RET* fusions, emphasizing the central role RET kinase activity plays in these cancers. Although selpercatinib and pralsetinib are generally well-tolerated, real-world surveillance identified small bowel edema, chylous effusions and erectile dysfunction as potential adverse effects of selpercatinib (Kalchiem-Dekel *et al.* 2022, Tsang *et al.* 2022, Matrone *et al.* 2024). Despite the success of these highly selective RET inhibitors, complete responses are seen in only 10% patients and the presence of residual disease allows for the development of treatment resistance.

## Primary resistance

Primary resistance to highly selective RET inhibitors is rare, occurring in around 2% of MTC patients on selpercatinib in clinical trials (Wirth *et al.* 2020, Hadoux *et al.* 2023*a*,*b*). A molecular cause for primary resistance to selpercatinib was reported for two patients with *RET* fusions, in whom pre-treatment plasma sequencing revealed *KRAS* mutations (G12D and G12V), the allele fractions of which increased on treatment (Rosen *et al.* 2022) (Supplementary Table). Interestingly, in each case, the *RET* fusion remained suppressed on treatment raising the possibility multiple tumor clones with distinct drivers (either *RET* or *KRAS*) might co-exist at the baseline and behave differently under selective pressure of RET inhibition (Rosen *et al.* 2022). In other cases, the cause of primary resistance to selpercatinib has not been established (Pitoia *et al.* 2024).

## Acquired resistance: on-target

**Figure 2:**
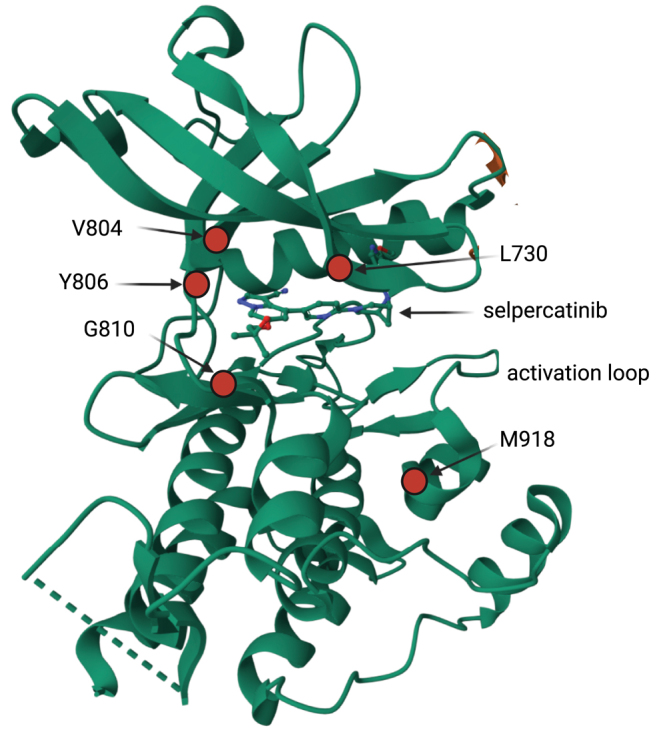
On-target RET resistance. Ribbon diagram of RET kinase–selpercatinib complex (7JU6:chain B) (Subbiah *et al.* 2021*a*). Key residues mediating on-target resistance to selpercatinib are shown as *red* dots: solvent front (G810), hinge (Y806), gatekeeper (V804) and roof (L730). The commonest codon mutated in MTC, *RET* M918, is also shown in proximity to the activation loop. Created with BioRender.com after importing structure from the Protein Data Bank (PDB).

On-target RET mutations causing resistance to selective kinase inhibitors occur at the solvent front, hinge region or roof of the ATP-binding pocket (Fig. 2). These mechanisms of RET resistance were foreshadowed by preclinical studies. Using random mutagenesis of *KIF5B-RET* vectors transduced into Ba/F3 murine B cells and treated with cabozantinib, lenvatinib or vandetanib, several *RET* kinase domain mutations were identified as pan-resistance markers including solvent front G810S and roof L730I (Liu *et al.* 2018), which would later be identified as mediating resistance to selpercatinib and pralsetinib.

The first clinical description of solvent front mutations was in a 61-year-old man with metastatic *KIF5B-RET* NSCLC progressing after carboplatin, pemetrexed, pembrolizumab and lenvatinib who commenced selpercatinib and had rapid clinical improvement until progression at 6 months and death at 7 months. Serial ctDNA analyses found *RET* G810S emerged some 3 months before clinical progression became apparent. Remarkably, tissue samples obtained at rapid autopsy were heterogeneous for distinct metastatic subclones containing G810R or G810S or G810C (Solomon *et al.* 2020). This report was extended by finding acquired mutations at *RET* G810 in three additional cases with *RET*-altered cancers progressing on selpercatinib: *CCDC6-RET* fusion NSCLC, *RET* fusion NSCLC and *RET* mutant MTC, respectively (Solomon *et al.* 2020) (Supplementary Table).

**Figure 3:**
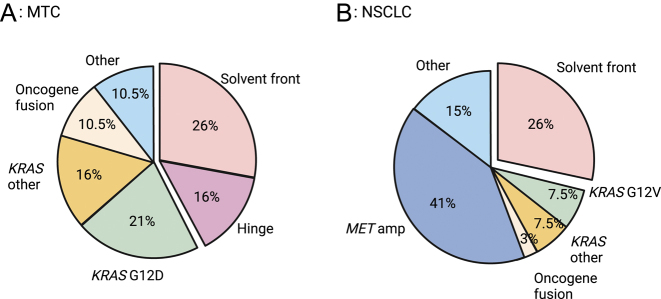
Range of mechanisms for resistance to RET inhibitors. On-target (solvent front and hinge) and off-target (*KRAS* mutations, oncogene fusion, MET amplification, ‘other’) resistance mechanisms are shown for (A) MTC and (B) NSCLC. Data derived from cases where molecular mechanism for resistance to highly specific RET inhibitor is reported (see Supplementary Table). Created with BioRender.com.

Since then, *RET* solvent front mutations have been reported in many other *RET*-altered NSCLC and MTC treated with either selpercatinib or pralsetinib, accounting for around 20–25% cases of acquired resistance (Lin *et al.* 2020, Subbiah *et al.* 2021*a*, Gainor *et al.* 2021*b*, Rosen *et al.* 2022, Hadoux *et al.* 2023*b*) (Fig. 3) (Supplementary Table).

Extensive experimental evidence supports mutation at RET G810 as a mechanism of resistance to selpercatinib. *RET* G810S was shown to evolve in a patient-derived xenograft model using a lung cancer sample with *CCDC6-RET* in mice treated orally with selpercatinib (Solomon *et al.* 2020). *In vitro* experiments confirmed *RET* G810S/R/C mutants were resistant to selpercatinib, pralsetinib, cabozantinib and vandetanib (Solomon *et al.* 2020, Subbiah *et al.* 2021*a*). BaF3 cells containing *KIF5B-RET* and subject to a random mutation library or prolonged culture with selpercatinib acquired *RET* V738A, Y806C/N and G810S; of note, V738A is outside coverage of clinical cell-free DNA assays and therefore may be an undetected cause of on-target resistance. *RET* G810 substitutions had minor effects on ATP affinity, consistent with direct inhibition of drug binding rather than increased kinase activity (Subbiah *et al.* 2021*a*). Structural modeling showed these acquired *RET* mutations caused steric hindrance to selpercatinib binding (Solomon *et al.* 2020, Subbiah *et al.* 2021*a*).

Rosen *et al.* (2022) extended the spectrum of on-target RET resistance describing a novel mutation in the hinge region (Y806C) when *in cis* with V804M; in combination, these mutations were shown to induce steric hindrance to selpercatinib binding. Intriguingly, the double V804M/Y806C mutation has been reported in an MEN2B-like family and shown *in vitro* to increase RET-transforming activity by 8–13-fold higher than that of single RET mutants (Iwashita *et al.* 2000). Aside from this unique case, and in contrast to the gatekeeper mutation *RET* V804M, which accounts for 26% cases of MEN2 (Elisei *et al.* 2019), available evidence suggests that no other naturally occurring *RET* mutants are intrinsically resistant to selpercatinib (Szymczak *et al.* 2023); IC_50_ *in vitro* is mildly higher for some mutants, such as *KIF5B::RET* and *RET* E632_L633del compared with *RET* M918T, but preclinical evidence suggests complete RET inhibition is achieved at concentrations of selpercatinib likely to be achieved *in vivo*, regardless of the underling *RET* mutation.

There may be differences in RET resistance to selpercatinib or pralsetinib; for instance, L730V/I mutations in the roof of the solvent front are resistant to pralsetinib but not apparently to selpercatinib (Shen *et al.* 2021) (Fig. 2). A preliminary analysis of pralsetinib resistance occurring in the ARROW trial reported these *RET* L730 mutations in ctDNA samples (Gainor *et al.* 2021*b*).

*RET* mutations mediating on-target resistance to highly specific inhibitors will be resistant to MKIs as well (Fancelli *et al.* 2021), emphasizing the urgent need to find new therapies to overcome this mechanism of resistance. This has so far proven challenging. Promising preclinical data have been reported for several compounds including TPX-0046, a potent RET/SRC inhibitor with preclinical potency against RET G810 solvent front mutations, which was in phase 1 testing in patients with advanced RET-altered solid tumors (NCT04161391) (Drilon *et al.* 2020*b*), and vepafestinib (TAS0953/HM06), a next-generation RET inhibitor, which inhibited *RET* variants at codons L730, V804 and G810 with a unique binding mode and additionally showed improved brain penetration in an intracranial model of RET-driven cancer (Miyazaki *et al.* 2023) and which is being investigated in an ongoing phase 1/2 clinical trial (NCT04683250).

## Acquired resistance: bypass

Bypass resistance is defined by progressive disease occurring despite ongoing suppression of RET and accompanied by the activation of one or more alternate oncogenic pathways. Indeed, most of the cases progressing on RET-selective inhibitors are driven by these off-target, RET-independent mechanisms of resistance (Lin *et al.* 2020, Hadoux *et al.* 2023*b*) (Fig. 3). Bypass resistance has been associated with the reactivation of the MAPK pathway *via* oncogene gain by amplification (*MET*, *KRAS*, *FGFR1* or *HER2*), activating mutations (*KRAS*, *HRAS*, *NRAS*, *BRAF* or *MAP2K*), fusion events (*NTRK* or *ALK*) or tumor suppressor loss (*CDKN2*) (Table 1) (Fig. 3).

**Table 1:** Mechanisms of resistance to RET inhibitors.

Target	Mechanism	Mutation	Resistant to	Susceptible to	References
**Primary**					
On-target	Gatekeeper: Steric hindrance	*RET* V804L/M	Cabozantinib, vandetanib	Selpercatinib, pralsetinib	Subbiah *et al.* (2018*a*,*b*), Wirth *et al.* (2019)
Bypass	Oncogene mutation	*KRAS* G12D/V	Selpercatinib	-	Rosen *et al.* (2022)
**Acquired**					
On-target	Gatekeeper: Steric hindrance	*RET* V804L/M	Cabozantinib, vandetanib	Selpercatinib, pralsetinib	Subbiah *et al.* (2018*a*,*b*), Wirth *et al.* (2019)
On-target	Solvent front: Steric hindrance	*RET* G810S/C/R/V	Selpercatinib, pralsetinib	-	Solomon *et al.* (2020), Lin *et al.* (2020), Subbiah *et al.* (2021*b*), Rosen *et al.* (2022), Hadoux *et al.* (2023*b*)
On-target	Hinge: Steric hindrance	*RET* Y806C (in cis with V804M)	Selpercatinib, pralsetinib	-	Rosen *et al.* (2022), Hadoux *et al.* (2023*b*)
On-target	Roof: Steric hindrance	*RET* L730I	Pralsetinib	-	Gainor *et al.* (2021*b*)
Bypass	Oncogene amplification	*MET*	Selpercatinib, pralsetinib	Crizotinib, capmatinib	Lin *et al.* (2020), Rosen *et al.* (2021), Zhu *et al.* (2021), Leite *et al.* (2023)
		KRAS	〃	-	Lin *et al.* (2020)
		*FGFR1*	〃	-	Rosen *et al.* (2022)
		*HER2*	〃	Trastuzumab	Vakkalagadda & Patel (2023)
Bypass	Oncogene activating mutation	*KRAS* G12A/R/V, G13D, A59del	Selpercatinib, pralsetinib	-	Rosen *et al.* (2022)
		*KRAS* G12D	〃	-	Hadoux *et al.* (2023*b*)
		*NRAS* G13E, Q61R	〃	-	Rosen *et al.* (2022)
		*BRAF*	〃	-	Rosen *et al.* (2022)
		*HRAS* A59T	〃	-	Hadoux *et al.* (2023*b*)
		*MYCN* P44L	〃	-	Hadoux *et al.* (2023*b*)
		*MAP2K* E102-I103 del	〃		Pishdad *et al.* (2024)
Bypass	Oncogene fusion	*NTRK3*	Selpercatinib, pralsetinib	Larotrectinib	Subbiah *et al.* (2021*d*)
		*ETV6::NTRK3*		Larotrectinib	Subbiah *et al.* (2024)
		*EML4::ALK*		Entrectinib	Subbiah *et al.* (2024)
Bypass	Tumor suppressor loss	*CDKN2A*, *CDKN2B*	Selpercatinib, pralsetinib	-	Porcelli *et al.* (2023)

*MET* amplification is a common off-target signaling pathway in NSCLC bypassing drivers such as *EGFR* mutations or *ALK* fusions (Engelman *et al.* 2007, Piotrowska *et al.* 2018, Dagogo-Jack *et al.* 2020, Schoenfeld *et al.* 2020). Similarly, *MET* amplification has been found in patients with *RET* fusion positive NSCLC progressing on selpercatinib or pralsetinib (Lin *et al.* 2020, Rosen *et al.* 2021, 2022, Zhu *et al.* 2021) (Fig. 3). In one case, the addition of crizotinib (a MET inhibitor) to selpercatinib extended treatment response by 10 months (Rosen *et al.* 2021). *In vitro* data showed *MET* overexpression-induced selpercatinib resistance, which could be overcome by crizotinib (Lin *et al.* 2020). Another case of selpercatinib resistance associated with *MET* amplification achieved durable response after adding capmatinib (Leite *et al.* 2023).

Subbiah *et al.* (2024) have recently reported a remarkable example of multiple bypass resistance mechanisms emerging on RET inhibition, several of which were targeted successfully with sequential combination therapy. A patient presenting with metastatic MTC associated with somatic *RET* D898_E901del was treated with selpercatinib after thyroidectomy and neck dissection; disease progression at 24 months associated with *ETV6::NTRK3* fusion was treated with larotrectinib in addition to selpercatinib; further disease progression after another nine months associated with *EML4::ALK* fusion was treated by switching from larotrectinib to entrectinib; unfortunately, further progression associated with *NTRK3* G623R ultimately led to treatment failure. This report also emphasized the utility of using liquid biopsy to detect these bypass mechanisms and guide personalized treatment changes. *NTRK3* fusion had previously been shown to induce resistance to selpercatinib in *KIF5B-RET*-transformed BaF3 cells, which was responsive to the combination of selpercatinib and larotrectinib (Subbiah *et al.* 2021*d*).

Similar success in targeting bypass mechanisms of RET inhibitor resistance was seen using trastuzumab to target *HER2* amplification (Vakkalagadda & Patel 2023).

Interestingly, *HRAS* mutations at Q61 and G13, which are the most frequent non-*RET* mutation in sporadic MTC (and mutually exclusive to *RET* mutations) (Xu *et al.* 2024), have not yet been described as a mechanism of resistance to RET inhibitors.

## Resistance due to other mechanisms

Although favorable CNS responses have been reported in patients treated with selpercatinib or pralsetinib (Drilon *et al.* 2020*a*, Gainor *et al.* 2021*a*), not all patients show response in the brain. A recent report demonstrated around one-third of patients with baseline brain metastases suffered from CNS progression while on therapy with selpercatinib (Subbiah *et al.* 2021*c*). Variable expression of ATP-binding cassette (ABC) transporters, especially the multidrug resistance protein 1 (MDR1, also known as P-glycoprotein encoded by *ABCB1*), is known to confer resistance to cytotoxic and targeted chemotherapy and may be particularly relevant for brain penetration (Robey *et al.* 2018). Animal models have shown P-glycoprotein (ABCB1/MDR1) and BCRP (ABCG2) limit brain accumulation of selpercatinib (Wang *et al.* 2021) and further research is required to determine whether this may account for some cases of RET inhibitor resistance.

## Limitations of existing data and areas for future research

Despite progress in understanding the mechanisms of RET resistance, many cases of both primary and acquired resistance remain unexplained (Rosen *et al.* 2022, Pitoia *et al.* 2024). There is clearly more to be learned about pathways to resistance and ways to block these. Fibroblast growth factor receptor (FGFR) signaling may play a role both as a driver of some thyroid cancers and as a mechanism of resistance to RET inhibition (Rosen *et al.* 2022, Sabbagh *et al.* 2024), and FGFR inhibition has been shown to mitigate resistance to a selective RET inhibitor in *CCDC6-RET* containing TPC1 cells and a transgenic zebrafish model (Raman *et al.* 2022). In MTC cell lines, pralsetinib resistance was found to be accompanied by aberrant activation of the Hedgehog/GLI pathway which was susceptible to mitigation by Hh/GLI inhibitors (Trocchianesi *et al.* 2023). Undoubtably, multiple mechanisms of resistance are likely to evolve and co-exist with prolonged treatment on selective inhibitors.

At this stage, it seems unlikely immune evasion represents a mechanism of resistance to RET inhibition. In a retrospective study of 74 patients with *RET*-rearranged lung cancers, both PD-L1 expression and tumour mutation burden (TMB) were low, and outcomes in 16 patients who had received immunotherapy were poor (Offin *et al.* 2019). Whether RET inhibition alters this immunologically cold phenotype is unknown.

The biological basis for determining whether a cancer develops on-target or bypass resistance remains unclear. In some cases, resistance-causing mutations may be preexisting (Lin & Shaw 2016, Rosen *et al.* 2021, 2022). Polyclonal resistance has also been observed: solvent front *RET* mutations co-occurring with *KRAS* G12 mutations have been reported in two cases, suggesting that independent clonal trunks had emerged on selpercatinib treatment (Rosen *et al.* 2022, Hadoux *et al.* 2023*b*). Although most cases of bypass resistance are associated with persistent suppression of *RET* (evidenced on liquid biopsy), in at least some cases the bypass resistance variant emerges in parallel with increasing *RET* allele frequency, and it is unclear whether this represents simply a passenger event to the bypass loop or loss-of-target engagement by RET inhibitors (Zhu *et al.* 2021, Lin *et al.* 2020). Whether upfront combination treatments might prevent acquired resistance needs further study. A recent report highlighted the utility of cabozantinib targeting simultaneously both MET and RET in a case of NSCLC in which acquired resistance to *MET* exon 14 skipping arose via emergence of a *RET* fusion on treatment with a MET-specific inhibitor (Torrado *et al.* 2024).

The rapid uptake of liquid biopsies has accelerated clinical translation of known mechanisms of resistance but is less sensitive than tissue biopsies to clonal heterogeneity and may impede discovery of the full range of genetic evolution under selective pressure of kinase inhibitors (Lin & Shaw 2016). Diagnostic accuracy of liquid biopsies depends on shedding of tumor-derived DNA (in turn, dependent on cancer type, extent and location of disease) and coverage and depth of the assay; whereas older assays for ctDNA had limited sensitivity for some *RET* variants and fusions (Allin *et al.* 2018, Ciampi *et al.* 2022), newer NGS assays have improved concordance with tissue testing and can detect *RET* mutations or fusions at very low allelic frequencies (Belli *et al.* 2021). Nevertheless, even using an ultra-deep sequencing targeted cancer gene panel, Rosen *et al.* (2022) detected *RET* alterations on liquid biopsy in 95% of *RET* mutant cases but only 74% of *RET* fusion cases, and in cases who had developed resistance, longitudinal plasma samples were able to detect a genomic mechanism of resistance in only 61% cases. Moreover, it is still crucial to know which genes are covered to properly interpret a ctDNA result; for instance, in the case of advanced MTC reported by Subbiah *et al.* (2024) mentioned above, an *ETV6::NTRK3* fusion was not covered on a liquid biopsy panel and only detected on tissue biopsy using a combined DNA/RNA-based NGS assay.

More broadly, there is no clear consensus on when and how to perform genetic reassessment when resistance to RET inhibition is suspected in clinical practice, particularly in resource-constrained healthcare – acknowledging responses of *RET*-altered cancers to highly selective inhibitors such as selpercatinib are often durable for years (Hadoux *et al.* 2023*a*). The European Society of Medical Oncology has published recommendations for methods to diagnose *RET* fusions and mutations at initial presentation, but this did not include guidance for re-sampling on diagnosis of progression on RET inhibitor therapy (Belli *et al.* 2021). The many research studies cited herein highlight the range of mechanisms by which resistance is acquired and also emphasize finding a result may not lead to change in management. Detection of a druggable target may be worthwhile for some patients but requires assays to cover a higher number of potential bypass oncogenes, whereas finding a solvent front or hinge *RET* mutation can be achieved using more focused sequencing but offers little solace in the absence of an efficacious second-generation RET inhibitor. A cost-effective approach may be to use targeted sequencing on biopsies of radiologically progressing lesions where possible, reserving higher coverage NGS assays when ‘hot-spot’ mutations (e.g., *RET* solvent front or *KRAS* G12X) are absent.

More data for acquired resistance come from lung cancer cases than from thyroid cancer cases at this stage (Supplementary Table). Moreover, clinicopathological risk factors associated with acquired resistance are yet unclear: specifically, whether specific tumor features (e.g., cancer type, Ki-67, tumor mutation burden, metastatic burden and duration of disease), pre-treatment with other therapies or duration and/or dose of selective inhibitor contribute most strongly to acquisition of resistance. A recent study reported outcomes from 46 patients with MTC on RET inhibitors, of whom 16 progressed and four died; notably, serial biopsies were available from some cases and showed increasing Ki-67 and more poorly differentiated histology as progressive disease emerged on treatment (Hadoux *et al.* 2023*b*).

## Conclusions

The discovery of the *RET* proto-oncogene and its association with cancer has led to the development of highly specific RET inhibitors, which have transformed the therapeutic landscape for *RET*-altered cancers, including thyroid and NSCLCs. This success is tempered by the emergence of resistance to these inhibitors with prolonged treatment; whether resistance is inevitable in every case remains to be seen. Molecular mechanisms have been defined for primary and acquired resistance, including on-target and bypass oncogenic activation. Strategies for preventing or treating resistance are beginning to be identified, particularly when bypass resistance is mediated by a druggable oncogene (e.g., *NTRK*, *ALK*, *MET* or *HER2*); in such cases, it is recommended to also continue RET inhibitor therapy. Conversely, solventfront mutations may be susceptible to second-generation RET inhibitors currently in development but account for only 20–25% cases of resistance. There is still an urgent need to find new therapies for patients who otherwise succumb rapidly once resistance develops. Future research will define optimal timing for starting targeted therapies, stratifying those at greatest risk of developing resistance, and targeting patient-specific resistance with next-generation RET inhibitors or combination therapies either up-front or sequentially.

## Supplementary materials



## Declaration of interest

The author declares that there is no conflict of interest that could be perceived as prejudicing the impartiality of the work.

## Funding

This work did not receive any specific grant from any funding agency in the public, commercial or not-for-profit sector.
