# Supplementary material for: Mechanisms of resistance to RET-directed therapies
Source: Endocr Relat Cancer. 2025 Jan 10;32(2):e240224. doi: 10.1530/ERC-24-0224 (PMC11798414; doi:10.1530/ERC-24-0224)
Supplement: Supplementary file 1 [file supplementary_materials.pdf]

Supplementary Table: 51 cases of resistance to RET inhibitors where mechanism reported

Patient	Tumour	RET	somatic/germline	Pretreatment	RET inhibitor	PFS (mo)	Mechanism of resistance	Discovered	Additional treatment	Ref
**Resistance to MKIs**										
49M	MTC	*RET* M918T	somatic	sorafenib	vandetanib, cabozantinib	60	*RET* V804M	plasma	selpercatinib	Subbiah et al 2018
44F	NSCLC	*KIF5B-RET*	somatic	chemotherapy, immunotherapy	alectinib	48	(brain)	na	selpercatinib	Subbiah et al 2018
42F	NSCLC	*KIF5B-RET*	somatic	chemotherapy, immunotherapy, TKIs	alectinib, vandetanib	13	*RET* V804L	tissue	selpercatinib	Wirth et al 2019
50M	MTC	*RET* V804M	germline		cabozantinib, vandetanib, lenvatinib	66	*RET* V804M	tissue	selpercatinib	Wirth et al 2019
**Resistance to highly selective RET inhibitors**										
61M	NSCLC	*KIF5B-RET*	somatic	carbo/pemetrexed/pembro/lenvatinib	selpercatinib	6	*RET* G810S/C/V/R	plasma		Solomon et al 2020
nr	NSCLC	*CCDC6-RET*	somatic	multiple	selpercatinib	10	*RET* G810S/C/V/R	tissue		Solomon et al 2020
nr	MTC	*RET* M918T	germline	Vandetanib, cabozantinib	selpercatinib	5	*RET* G810S	plasma		Solomon et al 2020
nr	MTC	*RET* M918T	somatic	Vandetanib	selpercatinib	4	*RET* G810S (and later G810C, Y806S/C)	plasma		Solomon et al 2020
nr	NSCLC	*CCDC6-RET*	somatic	mulitple MKIs	pralsetinib/selpercatinib	6.4	*RET* G810S	tissue		Lin et al 2020
nr	NSCLC	*KIAA1468-*	somatic	nr	pralsetinib	5.4	*MET* amp	tissue		Lin et al 2020

		RET; SLC24A1 (exon 6)-RET (exon 8)								
nr	NSCLC	*CCDC6-RET*	somatic	nr	selpercatinib	20	*RET* G810C	tissue		Lin et al 2020
nr	NSCLC	*KIF5B-RET*	somatic	chemoradiation, durvalumab, immunotherapy, MKI	selpercatinib	7.4	*MET* amp	plasma		Lin et al 2020
nr	NSCLC	*KIF5B-RET*	somatic	nr	pralsetinib	8	*MET* amp	tissue and plasma		Lin et al 2020
nr	NSCLC	*KIF5B-RET*	somatic	nr	selpercatinib	16.7	*KRAS* amp	tissue		Lin et al 2020
48F	NSCLC	*KIF5B-RET*	somatic	EBRT, pembrolizumab, carboplatin, pemetrexed	selpercatinib	7.5	*MET* amp	plasma	cabozantinib, capmatinib	Zhu et al 2021
49M	MTC	*RET* M918T, V804M/L	somatic	sorafenib, vandetanib, cabozantinib	selpercatinib	25	*RET* G810S/C (and Y806C/N)	plasma		Subbiah et al 2021a
66M	NSCLC	*CCDC6-RET*	somatic	carbo/pemetrexed etc	selpercatinib	18	*RET* G810C	plasma		Subbiah et al 2021a
36F	NSCLC	*EML4-RET*	somatic	multiple	selpercatinib	6.5	*MET* amp	tissue	crizotinib	Rosen et al 2021
48M	NSCLC	*KIF5B-RET*	somatic	pembrolizumab	selpercatinib	11	*MET* amp	tissue	crizotinib	Rosen et al 2021
69M	NSCLC	*KIF5B-RET*	somatic		selpercatinib	3	*MET* amp	tissue	crizotinib	Rosen et al 2021
61F	NSCLC	*KIF5B-RET*	somatic	pembrolizumab	selpercatinib	6	*MET* amp	tissue	crizotinib	Rosen et al 2021

62M	NEC	*KIF5B-RET*	somatic	carbo/etoposide and brain EBRT	selpercatinib	10	*KHDRBS1-NTRK3*	tissue		Subbiah et al 2021d
nr	MTC	*RET* M918T	somatic		selpercatinib	6	*RET* Y806C (+V804M), *KRAS* G12D/G13D	tissue/plasma		Rosen et al 2022
nr	PDTC	*CCD6-RET*	somatic		selpercatinib	8	?			Rosen et al 2022
nr	HGNEC	*TAF3-RET*	somatic		selpercatinib	primary	*KRAS* A59del/G12D	plasma		Rosen et al 2022
nr	NSCLC	*ERC1-RET*	somatic		selpercatinib	primary	*KRAS* G12V, NRAS *Q61R*	plasma		Rosen et al 2022
nr	NSCLC	*KIF5-RET*	somatic		selpercatinib	3	*KRAS* G12V	tissue/plasma		Rosen et al 2022
nr	NSCLC	*KIF5-RET*	somatic		selpercatinib	4	*MET* amp	plasma		Rosen et al 2022
nr	NSCLC	*KIF5-RET*	somatic		selpercatinib	4	*MET* amp	plasma		Rosen et al 2022
nr	NSCLC	*KIF5-RET*	somatic		selpercatinib	8	*RET* G810S, *KRAS* 12A/R, *HER2* amp	plasma		Rosen et al 2022
nr	NSCLC	*KIF5-RET*	somatic		selpercatinib	10	*NRAS* G13D	plasma		Rosen et al 2022
nr	NSCLC	*CCD6-RET*	somatic		selpercatinib	6	*FGFR1* amp	plasma		Rosen et al 2022
nr	Lung	*KIF5-RET*	somatic		selpercatinib	10	*RET* G810C	plasma		Rosen et al

	NEC									2022
35M	MTC	*RET* p.D898_E901	somatic	vandetanib	selpercatinib		?	tissue		Porcelli et al 2023
40sF	MTC	*RET* p.D898_E901	somatic	(surgery)	selpercatinib	24	*ETV6::NTRK3*	tissue	larotrectinib	Subbiah et al 2024
"	"		"	"	"	31	*EML4::ALK*	plasma	entrectinib	"
"	"		"	"	"		*NTRK3* p.G623R	plasma		"
65F	NSCLC	*KIF5B-RET*	somatic	carboplatin, penetrexed, pembrolizumab	selpercatinib	17	*ERBB2* (HER2) amp	tissue	trastuzumab	Vakkalagadda et al 2023
57F	NSCLC	*ISOC1-RET*	somatic	carboplatin, penetrexed, pembrolizumab	selpercatinib	30	*MET* amp	tissue	capmatinib	Leite et al 2023
nr	MTC	*RET* ms	nr	nr	selpercatinib		*KRAS* G12D	tissue		Hadoux J et al 2023
nr	MTC	*RET* ms	nr	nr	selpercatinib		*KRAS* G12D	tissue		Hadoux J et al 2023
nr	MTC	*RET* ms	nr	nr	pralsetinib		*KRAS* G12D	plasma		Hadoux J et al 2023
nr	MTC	*RET* ms	nr	nr	pralsetinib		*MYCN* P44L	plasma		Hadoux J et al 2023
nr	MTC	*RET* ms	nr	nr	selpercatinib		*RET* G810	plasma		Hadoux J et al 2023
nr	MTC	*RET* ms	nr	nr	pralsetinib		*HRAS* A59T	plasma		Hadoux J et al 2023

nr	MTC	*RET* ms	nr	nr	selpercatinib		*RET G810S, KRAS* G12D, G13_V14insG	plasma		Hadoux J et al 2023
nr	MTC	*RET* ms	nr	nr	pralsetinib		*KRAS* G12A	plasma		Hadoux J et al 2023
nr	MTC	*RET* ms	nr	nr	pralsetinib		*RET* Y806C	plasma		Hadoux J et al 2023
nr	MTC	*RET* ms	nr	nr	selpercatinib		*RET* Y806C	plasma		Hadoux J et al 2023
nr	MTC	*RET* ms	nr	nr	selpercatinib		*FGFR2-ATAD1*	plasma		Hadoux J et al 2023
nr	MTC	*RET* ms	nr	nr	selpercatinib		*KRAS* p.Q61L, G12R; *NRAS* Q61K and Q61R*;* *ALK* fusions*;* BRAF	plasma		Hadoux J et al 2023
70F	NSCLC	*CCD6-RET (acquired)*	somatic	Multiple	selpercatinib	primary	*MET* exon 14 skip	tissue	cabozantinib	Torrado et al 2024
50M	NEC	*KIF5B-RET*	somatic	vandetanib	selpercatinib	24	*MAP2K1* E102-I103 del	tissue		Pishdad et al 2024
